# Comprehensive survey of conserved RNA secondary structures in full-genome alignment of Hepatitis C virus

**DOI:** 10.1038/s41598-024-62897-0

**Published:** 2024-07-02

**Authors:** Sandra Triebel, Kevin Lamkiewicz, Nancy Ontiveros, Blake Sweeney, Peter F. Stadler, Anton I. Petrov, Michael Niepmann, Manja Marz

**Affiliations:** 1 RNA Bioinformatics and High-Throughput Analysis, Friedrich Schiller University Jena, 07743 Jena, Germany; 2 European Virus Bioinformatics Center, Friedrich Schiller University Jena, 07743 Jena, Germany; 3 European Molecular Biology Laboratory, Wellcome Genome Campus, European Bioinformatics Institute, Hinxton, Cambridge, CB10 1SD UK; 4 Bioinformatics Group, Institute of Computer Science, and Interdisciplinary Center for Bioinformatics, University Leipzig, 04107 Leipzig, Germany; 5 Riboscope Ltd., Cambridge, CB1 1AH UK; 6 Institute for Biochemistry, Justus-Liebig-University Giessen, 35392 Giessen, Germany; 7 Leibniz Institute on Aging-Fritz Lipmann Institute, 07745 Jena, Germany; 8 German Center for Integrative Biodiversity Research (iDiv), 04103 Leipzig, Germany; 9 Michael Stifel Center Jena, Friedrich Schiller University Jena, 07743 Jena, Germany; 10 Cluster of Excellence Balance of the Microverse, Friedrich Schiller University Jena, 07743 Jena, Germany

**Keywords:** Hepatitis C virus, Full-genome alignment, RNA secondary structure prediction, Computational biology and bioinformatics, Evolutionary biology, Coevolution, Evolutionary genetics

## Abstract

Hepatitis C virus (HCV) is a plus-stranded RNA virus that often chronically infects liver hepatocytes and causes liver cirrhosis and cancer. These viruses replicate their genomes employing error-prone replicases. Thereby, they routinely generate a large ‘cloud’ of RNA genomes (quasispecies) which—by trial and error—comprehensively explore the sequence space available for functional RNA genomes that maintain the ability for efficient replication and immune escape. In this context, it is important to identify which RNA secondary structures in the sequence space of the HCV genome are conserved, likely due to functional requirements. Here, we provide the first genome-wide multiple sequence alignment (MSA) with the prediction of RNA secondary structures throughout all representative full-length HCV genomes. We selected 57 representative genomes by clustering all complete HCV genomes from the BV-BRC database based on k-mer distributions and dimension reduction and adding RefSeq sequences. We include annotations of previously recognized features for easy comparison to other studies. Our results indicate that mainly the core coding region, the C-terminal NS5A region, and the NS5B region contain secondary structure elements that are conserved beyond coding sequence requirements, indicating functionality on the RNA level. In contrast, the genome regions in between contain less highly conserved structures. The results provide a complete description of all conserved RNA secondary structures and make clear that functionally important RNA secondary structures are present in certain HCV genome regions but are largely absent from other regions. Full-genome alignments of all branches of *Hepacivirus C* are provided in the supplement.

## Introduction

Hepatitis C virus (HCV) is a major public health concern that affects an estimated 71 million people globally^1^ causing liver cirrhosis and hepatocellular carcinoma. The virus is a member of the species *Hepacivirus C* and the *Flaviviridae* family, which also includes Dengue, Zika, and yellow fever viruses. HCV is primarily transmitted through blood-to-blood contact, with injection drug use being the most common mode of transmission. It can also be transmitted through unsafe medical procedures, blood transfusions, and from mother to child during childbirth^2–4^.

HCV is a single-stranded positive-sense RNA virus. The HCV genome is approximately 9.6 kb in length and organized into a single open reading frame (ORF) flanked by two untranslated regions (UTRs) at the $5^{\prime }$ and $3^{\prime }$ ends. The ORF encodes a single polyprotein precursor that is co- and post-translationally cleaved by host and viral proteases into structural (C, E1, E2, p7) and non-structural proteins (NS2, NS3, NS4A, NS4B, NS5A, NS5B), which play critical roles in viral replication and pathogenesis^5–7^. The HCV genome is known to form several RNA secondary structures^8–16^. The $5^{\prime }$ UTR contains four structural domains (I–IV). Three of those (II–IV) form a highly structured RNA region called the internal ribosome entry site (IRES), which is responsible for controlling the translation of the viral polyprotein. The IRES allows HCV to circumvent the host cell’s cap-dependent translation initiation mechanism, which is commonly used by cellular mRNAs. The region downstream of SL I including alternative SL II structures^17^ contains binding sites for microRNA-122 (miR-122) involved in translation, replication, and RNA stability^10,18–21^. The $3^{\prime }$ UTR contains several RNA structures that are involved in regulating viral RNA replication and translation, including a hypervariable region (HVR), a poly-U/UC tract, and a highly conserved RNA secondary structure called X-tail. The X-tail is a conserved RNA stem-loop structure that serves as a binding site for host proteins and is involved in viral replication and translation^10,14^. Apart from the structural motifs located in the UTRs, HCV showcases RNA secondary structures within its coding region, such as the *cis*-replication element^8–11,16,22,23^.

The HCV genome is highly variable due to the error-prone nature of its NS5B replicase^24,25^ and generates a cloud of ‘quasispecies’ that covers a huge sequence space that allows the virus to adapt to changing host environments and escape the immune system^26^, with eight genotypes and 93 subtypes identified to date, and sequence diversity of approximately 30% between genotypes^27,28^, see Fig. S1. Its genome contains several complex RNA structures that are critical for viral replication and pathogenesis. Thus, understanding the structure and function of these RNA elements is crucial for the development of effective treatments for HCV infection.

Previous studies have predicted conserved RNA secondary structures in important parts of the HCV genome by different approaches. These studies either analyzed mainly the $5^{\prime }$ and $3^{\prime }$ UTRs as well as the end of the NS5B coding region^10,16^, or they focused on certain HCV genome hotspot regions in the coding regions using covariance analysis with genotype 2 sequences and extending this to other genotypes^11^. Another study confined the prediction of RNA secondary structures in a full-length genome to the isolates JFH-1 (genotype 2a), H77c (genotype 1a), and Con1 (genotype 1b)^9^. The above studies provided important information on conserved RNA secondary structures and their functions in certain HCV RNA genome regions. However, up to the present, a complete survey of all conserved RNA secondary structures in the full length HCV across all genotypes is missing. Therefore, we filtered and manually refined a set of 2549 HCV full-length genome sequences that fully represent the phylogenetic diversity of all known HCV isolates. From this set, 57 HCV RNA genome sequences were selected that represent the complete phylogenetic tree’s sequence space of HCV genomes.

The in silico calculation of full-genome alignments for viral sequences, coupled with the prediction of RNA secondary structures, presents a multifaceted challenge in computational biology. Viral genomes exhibit high genetic diversity, characterized by rapid mutation rates and the presence of insertions and deletions. The complexity for the construction of a multiple sequence-secondary structure alignment (MSSSA) lays at $O(m\cdot n^6)$ and is therefore, not computationally feasible. For current construction of MSSSAs, the genomes have to be divided into smaller subsequences for a reliable prediction. Addressing these challenges is pivotal, as such alignments provide crucial insights into the molecular evolution of viruses and their structural-functional relationships. In this context, the development of robust computational methodologies is essential to advance our understanding of viral biology and host-virus interactions.

In this study, we present a full-genome alignment of HCV coupled with RNA secondary structure annotation. The alignment was generated using a semi-automated approach and underwent curation led by experts in the field of HCV and its associated structural elements. Beyond established structures, our study reveals previously unrecognized RNA secondary structures, predicted through computational methods, that exhibit conservation across HCV genomes. Moreover, the results of our alignment—using sequences covering the complete phylogenetic sequence space of HCV isolates—suggest that our predictions likely cover virtually all possible conserved RNA secondary structures.

## Material and methods

### Data

We downloaded 2,606 HCV genomes (June 01, 2023) from the BV-BRC database^29^. To ensure the quality of the data, we filtered the genome status ‘complete’ and excluded the host group ‘lab’. Notably, despite the genome completeness filter, the majority of entries of this data set (80.5%) contain incomplete genomes, lacking the UTRs. Among these, 20 genomes were excluded from the analysis due to their sequences containing 10% or more ‘N’s. After identifying duplicated genomes, the data set was refined to a total of 2549 genomes. Both the original data set and the pre-filtered data set are included in the Supplementary Files F1 and F2 in Fasta (.fasta) format.

### Finding representative genomes

We performed clustering of the pre-filtered data set (2549 genomes) based on k-mers to select sequences representing the data set. After calculating the k-mer profiles of the input sequences, we performed a dimension reduction by principal component analysis (PCA) followed by clustering using HDBSCAN v0.8.27^30^. HDBSCAN resulted in 36 representative genomes which were selected for further analysis, as this method provided comprehensive coverage of the genome information space. For comparison, we clustered sequences with five algorithms: cd-hit-est^31,32^, MMSeqs2^33^, sumaclust^34^, vclust^35^, and HDBSCAN^30^ (see Table S1). The workflow is implemented in ViralClust^36^. Despite all filters applied, partial genomes are present in the data set, and thus, the cluster representatives calculated by HDBSCAN did too. We removed six sequences manually from the set of representative genomes because they were too short (less than 1000 nt: MK468966, MK468983, MK469005, MK468990, and OM896954), or were not related to a functional polyprotein (EU862828). However, it was necessary to manually enlarge our set of representative genomes to fully display the entire spectrum of HCV samples. Utilizing the phylogenetic tree of the pre-filtered data set, see Fig. S1 and Supplementary File F3, we added 20 genomes representing outliers or subtrees not covered by the clustering results (black squares in Fig. S1). Additionally, for comparison to known strains, we added the NCBI RefSeq genomes of HCV (NC_038882, NC_004102, NC_009823, NC_009824, NC_009825, NC_009826, NC_009827, NC_030791) to our final set of representative genomes^37^. We removed one sequence (OM896952) because of high redundancy with NC_009824. Finally, a total of 57 representative genomes covering all eight genotypes of HCV were selected, see supplementary file F4. About 50% of the representative HCV genomes (27) contain the UTRs (see supplementary information subsection “Genome completeness of representative genomes”). The genome length of the selected genomes ranges from 9036 nt (MN164872.1) to 9711 nt (NC_009823.1).

### Multiple sequence alignment and RNA secondary structure prediction

The 57 representative genomes served as input for alignment construction. We computed an initial multiple sequence alignment (MSA) using MAFFT v7.520^38^ to identify highly conserved regions, which served as ‘anchors’ for further steps. Anchors are defined as segments in the MSA, requiring a minimum length of 10 nucleotides, and exhibiting an average Shannon entropy value lower than 0.1. Subsequently, we focused our analysis on the subregions between these anchors, utilizing LocARNA v2.0.0^39^. The subregions were then merged into one MSA, followed by an RNA secondary structure prediction of the full-genome alignment with a window-based approach. These steps are implemented in VeGETA using Python v3.7.12^40,41^. Additionally, we intensively examined and slightly curated the alignment manually. Finally, we added the annotation of conserved RNA secondary structures described in the literature as well as novel ones. Based on the nucleotide alignment, we constructed the protein alignment of the representative genomes.

## Results and discussion

In this study, we present a comprehensive analysis of all conserved RNA secondary structures that occur in the complete sequence space represented by the phylogenetic tree of all HCV isolates (see Fig. S1). Thus, the results are supposed to provide a complete description of RNA secondary structures that may be advantageous for the viral life cycle.

It is known that functionally important RNA secondary structures not only occur in the untranslated regions of mRNAs but also in the coding regions^42^. A variety of molecular mechanisms can be envisioned to be employed by RNA secondary structure elements. RNA secondary structure elements in the protein-coding region can be used for influencing the translational outcome of a given RNA^43^, for example by inducing a ribosome frameshift or termination reinitiation. Specific RNA elements can be used for packaging selectively one RNA while another longer RNA species is excluded from packaging by translational inactivation^44^. A translating ribosome may also displace proteins from an RNA secondary structure element of the RNA and by that induce a kind of ‘burn after reading’ degradation of the RNA^45^. Thus, it is important to identify those RNA secondary structures that have been selected for their function from the available sequence space produced by the error-prone replicases of RNA plus strand viruses.

### Basic statistics of the full-genome alignments

Our nucleotide-based alignment contains 57 representative HCV genomes including all eight genotypes. The alignment spans 9831 residues and 23 061 gaps, averaging approximately 405 gaps per sequence. Approximately 8.5% of the sequences within our alignment contain non-ACGU characters, highlighting sequence variations that may have functional significance. Half of the sequences (28/57) exhibit a full $5^{\prime }$ UTR; four sequences lack the $5^{\prime }$ UTR entirely; 12 sequences are deficient in both stem-loops I (SL I) and II (SL II) in the $5^{\prime }$ UTR, and 13 sequences lack only SL I. For the $3^{\prime }$ UTR, only 11 sequences display a complete X-tail structure; three sequences show partial X-tail formations, all others lack the $3^{\prime }$ UTR. The seed sequence (ACACUCC) of the first miR-122 binding site of the $5^{\prime }$ UTR directly downstream of the SL I is present in all 32 sequences covering that region; the second miR-122 binding site (CACUCC) directly downstream is present in 37/40 sequences, and one other sequence contains CGCUCC which would also allow miR-122 binding by G-U base pairing. In the $3^{\prime }$ UTR, the miR-122 seed sequence ACACUCC is contained in 41/44 sequences, in contrast, three of the sequences in genotypes 6 and 8 do not contain this site.

We added additional information to our nucleotide alignment: (1) gene annotations (shown as annotation line #=GC Annotation in the stk file); (2) the F/ARFP frameshift (notated with ‘f’ in the Annotation line in the stk file); (3) the RNA secondary structures (including pseudoknots) documented in the literature, along with alternative structural configurations (see Table 1); (4) incorporated in-silico predicted novel RNA secondary structures, providing a comprehensive view of potential conformations within the HCV genome.

**Table 1:** Conserved RNA secondary structures (SS) in HCV genomes, along with their corresponding Rfam model IDs (if available)^46,47^.

RNA SS	Genomic region	Alignment position		JFH-1 position		Rfam v12	Rfam v14.10
		S	E	S	E		
SL I	$5^{\prime }$ UTR	13	28	5	19	RF00061	RF00061$^{\circ }$
SL II	$5^{\prime }$ UTR	52	126	43	117	RF00061	RF00061$^{\circ }$
SL III	$5^{\prime }$ UTR	133	334	124	322	RF00061	RF00061$^{\circ }$
SL IV	$5^{\prime }$ UTR/C	346	360	334	348	RF00061	RF00061$^{\circ }$
SL V	C	400	435	388	423	RF00620	RF00620$^{\circ }$
SL VI	C	439	519	427	507	RF00620	RF00620$^{\circ }$
**SL 562**	C	574	595	562	582		
SL 588	C	601	678	588	665		RF04220
SL 669	C	684	761	671	748		RF04221
J 750	C	762	839	749	826		RF04219
SL 833	C	846	869	833	856		RF00***
SL 1412	E1	1430	1467	1414	1451		RF04305
**SL 1850**	E1	1881	2013	1850	1982		
**SL 2313**	E1	2356	2433	2313	2390		
SL 2531	E2	2 574	2591	2531	2548		RF04308
SL 2549	E2/p7	2592	2636	2549	2592		RF04308
**SL 3308**	NS2/NS3	3352	3644	3308	3600		
**SL 3844**	NS3	3888	3952	3844	3908		
**SL 4005**	NS3	4049	4099	4005	4056		
**SL 4214**	NS3	4258	4223	4214	4279		
**SL 4527**	NS3	4571	4591	4527	4547		
**SL 4621**	NS3	4665	4725	4621	4681		
**SL 4691**	NS3	4735	4788	4691	4744		
**SL 5016**	NS3	5060	5125	5016	5081		
**SL 5128**	NS3	5172	5249	5128	5205		
**SL 5357**	NS4A	5401	5448	5357	5404		
**SL 5647**	NS4B	5691	5777	5647	5733		
**SL 6027**	NS4B	6071	6197	6027	6153		
**SL 6270**	NS5A	6314	6410	6270	6366		
**SL 6371**	NS5A	6415	6446	6371	6402		
**SL 6530**	NS5A	6574	6689	6530	6645		
**SL 7516**	NS5A	7584	7603	7516	7535		
**SL 7536**	NS5A	7604	7702	7536	7634		
**SL 7816**	NS5B	7893	7905	7816	7828		
J 7880	NS5B	7959	8073	7882	7996		RF00***
SL 8001	NS5B	8077	8126	8000	8049		RF04306
**SL 8075**	NS5B	8152	8174	8075	8097		
**SL 8299**	NS5B	8376	8396	8299	8334		
SL 8670	NS5B	8722	8803	8645	8726		RF04307
5BSL1	NS5B	9194	9231	9117	9154		RF04218
5BSL2	NS5B	9276	9335	9198	9257	RF00468	RF00468$^{\circ }$
5BSL3.1	NS5B	9361	9406	9283	9328	RF00260	RF00260$^{\circ }$
5BSL3.2	NS5B	9410	9455	9332	9377	RF00260	RF00260$^{\circ }$
5BSL3.3	NS5B	9467	9497	9389	9419	RF00260	RF00260$^{\circ }$
SL IV	NS5B	9505	9533	9427	9452	RF00469	RF00260$^{\circ }$
X-tail	$3^{\prime }$ UTR	9727	9826	9579	9678	RF00481	RF00481$^{\circ }$

We updated and merged six Rfam models of v12 into five Rfam models (v14.10); confirmed the conservation of a total of 16 previously predicted RNA secondary structures throughout the phylogenetic tree (available in Rfam v14.10); and added further 23 novel conserved RNA families into Rfam (bold font). $\circ$ indicates that the existing Rfam model was updated with this publication. We named novel structures according to their position in the genome JFH-1 (S—Start; E—End). ***Rfam models will follow in the near future.

The protein alignment of the 57 representative genomes encompasses a total of 3017 residues, with an average of 37 gaps (2136 gaps in total). A minor proportion of not characterized amino acid characters (0.0015%) accounting for a total of 262 occurrences, can be found in the alignment. These ‘X’s are based on ‘N’s in the sequences downloaded from NCBI. We provide the nucleotide, protein, and combined alignments in the supplementary material with several formats, such as Stockholm (stk), ClustalW (aln), and Fasta (fasta) (see Files F5 and Files F7), which can be conveniently visualized using tools such as ClustalX^48,49^, Jalview^50^ or Emacs RALEE mode^51^. We have visualized the possible color codes available in Emacs RALEE mode in Fig. S5 based on the examples SL V and SL VI.

### Alignment confirms previously annotated RNA secondary structures

**Figure 1:**
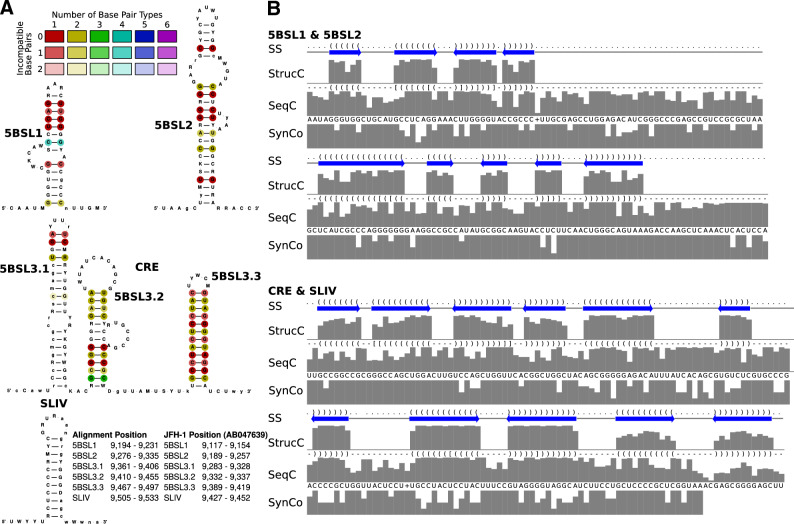
Known RNA secondary structures in the downstream NS5B coding region including the *cis*-replication element (CRE, 5BSL3.2) which are relevant for replication control. (**A**) RNA secondary structures are colored by the number of base pair types illustrating the extent of covariations in double-stranded regions. The SL IV (or 5BSL3.4) contains the NS5B stop codon in the apical loop. The structure was visualized using R2DT^59^. The nucleotide sequence shows the most informative sequence (IUPAC code) calculated by RNAalifold^60^ based on the alignment. Lowercase letters indicate gaps in the alignment column. (**B**) RNA secondary structure dot-bracket annotation (SS), structure consensus (StrucC), sequence consensus (SeqC), and the fraction of nucleotides used from synonymous codons (SynCo). Thereby, a low value indicates that only a few nucleotide(s) out of all nucleotides possible for synonymous codons are actually used by the different HCV isolates, indicating a high degree of primary sequence conservation which goes beyond the requirements of the coding sequence. This provides evidence that a conserved functional RNA element may overlap with the coding sequence. Alignments shown in Figs. S12 and S13.

**Figure 2:**
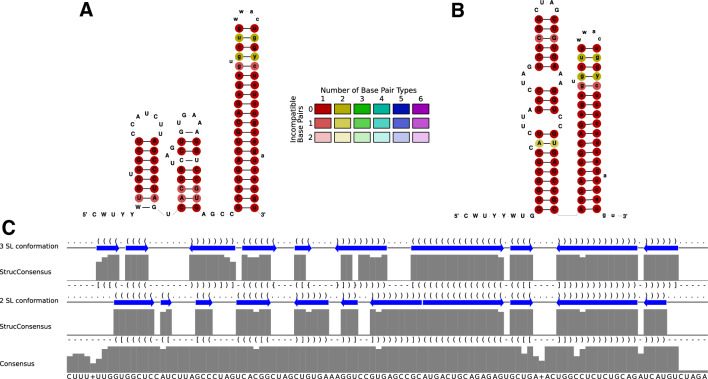
The two alternative structures of the highly conserved $3^{\prime }$ X region of the $3^{\prime }$ UTR. (**A**) The conformation with the three stem-loops SL 1, 2, and 3. In this form, the apical loop of SL 2 can make a long-range interaction (LRI) with the apical loop of the CRE/5BSL3.2 (‘kissing loop’ interaction). Among the selected sequences, only 11 isolates had a complete $3^{\prime }$ UTR sequence (please see the additional alignment of only $3^{\prime }$ X sequences in Fig. S14 and F9). (**B**) The conformation with SL 2 and 3 restructured to form the DLS that is speculated to be involved in HCV RNA genome dimerization^61–63^. (**C**) Additional sequence and RNA secondary structure features of the consensus of the 11 isolates, with two alternative dot-bracket outputs. As in parts of the $5^{\prime }$ UTR, the strong conservation of the primary sequence indicates that both overlapping structures shown in (**A**) and (**B**) may be functionally important, thereby limiting the extent of possible covariations in the RNA secondary structure regions of each conformation.

The full-genome alignment of HCV genomes reveals the presence of well-characterized RNA secondary structures, see Table 1, that are consistent with the existing literature^9–11,18^. All structures, including the long-range interactions and the genome circularization presented by Fricke et al.^10^, are annotated in our alignment. Therefore, the above alignment is validated by its prediction of these structures which had been demonstrated to be functional *cis*-elements involved in the regulation of HCV RNA translation and replication. These known structural elements include the highly conserved IRES (see Figs. S2 and S3), which plays a crucial role in HCV translation initiation^10,18^, as well as stem-loop structures within the core coding region (see Fig. S6). Additionally, the interaction of the sequence in the left base of SL VI in the core coding region with the single-stranded region between SL I and SL II in the $5^{\prime }$ UTR was shown; this interaction had been demonstrated to act inhibitory on translation^52–54^, while this inhibition can be relieved by microRNA-122 binding to the region between SL I and SL II^55,56^ (see S4). In the NS5B coding region (see Fig. 1 and Figs. S9–S13) the *cis*-replication element (CRE, composed of 5BSL3.1, 5BSL3.2, 5BSL3.3) and the highly conserved X-tail in the $3^{\prime }$ UTR (both conformations) (see Fig. 2 and Fig. S14) were identified. Moreover, we predicted several conserved RNA secondary structures in the coding region of HCV genomes (see Fig. 1, Figs. S6, S9, S10, S11, S12, and S13) in agreement with the literature^9–11^. The identification of these known structures in the core coding region^57,58^ and in the NS5B region^9,11^ not only validates the accuracy of our alignment but also underscores their functional importance in HCV biology: their conservation across different HCV genotypes further highlights their critical roles in viral replication, translation, and infection.

The CRE/5BSL3.2 and the 5BSL3.3 are important for HCV replication^22,23^. These functional aspects are reflected by the high conservation of the 5BSL3.2 and 3.3 (see Fig. 1 and Fig. S13), not only in their RNA secondary structure regions but also in their single-stranded loops and bulges, which are conserved beyond coding sequence requirements, since only selected nucleotides are actually used from those possible in synonymous codons (see Fig. 1 and Fig. S13). Likely, in the early phase after HCV infection, the CRE apical loop can interact with the SL 2 of the $3^{\prime }$ X region by forming a ‘kissing loop’ interaction^64^, and this interaction may stabilize its SL 2, SL 3 conformation^65,66^. At a time when sufficient amounts of NS5B replicase have been translated, NS5B can bind the CRE/5BSL3.2 and 5BSL3.3^23,67^, thereby disabling the CRE—SL 2 interaction and allowing refolding of the SL 3 and SL 2 in the $3^{\prime }$ X region to form the overlapping dimerization linkage sequence (DLS)^61,62^. Binding of NS5B to the CRE then is supposed to be involved in starting RNA minus strand synthesis at the HCV RNA $3^{\prime }$ end, whereas the role of the DLS and its putative role in dimerization of the full-length HCV genome in this process is not yet fully understood. These functional aspects in turn validate our alignment approach for identifying functionally important RNA secondary structures.

Similar constraints may apply to the region including SL II and the preceding sequence between SLs I and II is highly conserved in the primary sequence due to two alternative conformations that fulfill different tasks in the viral life cycle^68^. The classical conformation of SL II allows binding of two complexes of miR-122 with Argonaute (AGO) protein to the single-stranded region upstream of SL II and has roles in HCV genome replication^19^, promoting translation^20^ and stabilization of the genome against nucleolytic degradation^21^. The alternative conformation SL II$^{alt}$^17^, however, appears to have a role in HCV assembly^68^. We also confirmed the SL IIId and its conserved alternative form SL IIId* which showed up previously^10^ in the IRES (see Figs. S2 and S3). This alternative SL IIId* is predicted to be slightly more stable than the classical SL IIId. We can only speculate if this alternative SL IIId* represents a structure that may be important in the IRES when not bound to ribosomes (an additional discussion of this aspect can be found in the Supplementary Materials Subsection ‘Alignment confirms previously predicted RNA secondary structures—Additional Information’).

### Improvements to Rfam virus families

**Figure 3:**
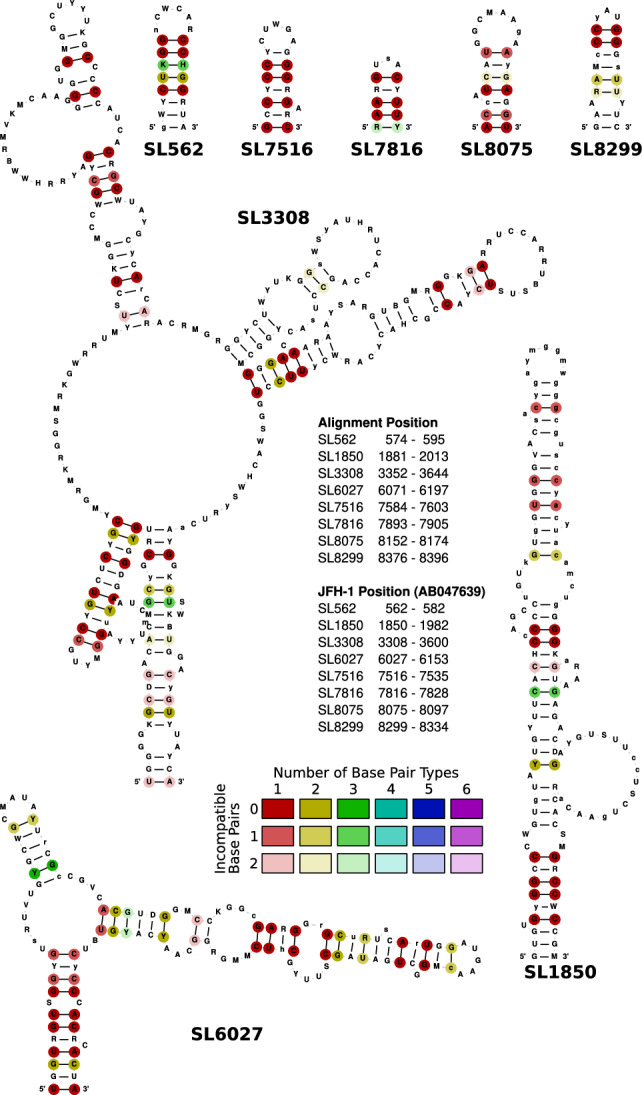
Eight novel selected conserved RNA secondary structure candidates from coding regions of the HCV alignment. RNA secondary structures are colored as in Fig. 1A.

We improved the models in the Rfam database^46,47^ (see Table 1) to ensure comprehensive coverage of the entire phylogenetic clade of the HCV sequences. In total, we identified 39 conserved structural regions across the HCV genomes, of which 23 were novel. We updated the six Rfam families from release 12 to nine families in release 14.10, of which all are validated by the literature. The HCV Rfam families were reviewed for covariance support with R-scape^69^. Only a small number of base pairs (1–3) exhibited covariance support in each family, and this consistency was observed across families of both non-coding and coding sequences/regions. The remaining 30 structured regions will be used to create additional Rfam families in future releases. In the following, we compare the five models to the previously well-described models from Rfam v12: (1) We reduced the IRES model (RF00061) from 79 to 51 sequences, spanning 356 nucleotide positions in the alignment (previously 413). The new model includes now SL I (from 30 HCV genomes) and SL II (covered by 41 genomes), which was absent due to sequencing problems of the very $5^{\prime }$ genome end in previous times. Importantly, the new model includes now SL IV from 51 HCV genomes, which is located at the transition from $5^{\prime }$ UTR to core gene, containing the start codon of the polyprotein. (2) The SL V and SL VI model (RF00620) was updated from 36 sequences to 56 (MK548369 excluded because of non-ACGU characters) and now spans an alignment length of 136 positions. The previous model contained 153 alignment positions, indicating a major reduction of gaps in the novel alignment. SL V was reduced by one base pair and SL VI by three base pairs. (3) The 5BSL2 model (RF00468) has now been reduced from 110 to 57 genomes. This measurement allows us to not compose a bias towards closely related, highly over-represented sequences. Our alignment expands the stem-loop by four base pairs. (4) The CRE model (RF00260) is now represented by all 57 selected genomes (previously 52). Only 5BSL3.2 of the CRE has been included in the old model, therefore the new model spans now 183 nucleotide positions (instead of 51 nucleotides) including the complete CRE (5BSL3.1, 5BSL3.2, and 5BSL3.3) and SL IV. (5) The model of SL IV (RF00469), located at the transition from NS5B gene to $3^{\prime }$ UTR, containing the stop codon of the polyprotein, comprised 110 sequences. This structure is now naturally merged into model RF00260. (6) Lastly, the X-tail model (RF00481) now contains only 11 sequences, the old model contained 22 HCV genomes. However, the old model only contained sequences of genotype 1–3. Therefore, although the total number of sequences has been reduced, the variety of the X-tail has been enlarged by our new model and is now spanning genotypes 1–6. We expanded SL I by one base pair. In total, we were able to predict 16 structural elements in the entire phylogenetic tree of HCV that had been previously confirmed^10,11,16^, see Figs. 1, 2, Figs. S2, S6, S7 and S9. We added these and the 23 novel HCV RNA secondary structures to Rfam v14, see Fig. 3 and Fig. S15. An information page for all HCV models in Rfam is provided at the following link: https://rfam.org/viruses/hcv.

### New RNA secondary structure element candidates

Our analysis revealed several novel RNA secondary structures in the coding region using our *in silico* prediction method (see Fig. 3 and Fig. S15). We selected novel RNA secondary structures based on their (1) conservation at the sequence and structural level in the representative sequences regarding compensatory mutations and (2) the use of synonymous codons especially in the hairpin region. We predicted 23 novel candidates to likely be functional elements due to their RNA secondary structure conservation which shows compensatory mutations despite being placed in the coding region, including the restricted use of synonymous codons (Fig. 3 and Fig. S15). SL 562 (see Fig. 3) is one example for a novel predicted short hairpin, which shows compensatory mutations despite being placed in the coding region of the core protein. SL 562 is represented in all 57 sequences highly conserved and structured.

At alignment pos. 7582 (SL 7516, see Fig. 3; and JFH-1 pos. 7516), an RNA secondary structure element with a seven base pair stem and a five nucleotide loop is predicted to be conserved that shows good conservation in the StructConsensus, Consensus as well as in the low number of exchanges in synonymous codons, indicating conservation beyond coding sequence requirements (see Fig. 3 and Fig. S16). In these terms, this newly predicted element is better conserved than the J 7880 element which was shown to be functional in early HCV replication^9^, suggesting that this new element may have functional importance, even though the degree of its conservation does not fully reach that of the CRE. Similarly, newly predicted RNA secondary structures at positions 7893 (SL 7816, 7816 in JFH-1), pos. 8152 (SL 8075, 8075 in JFH-1), and pos. 8376 (SL 8299, 8299 in JFH-1) are well conserved and are good candidates for functional RNA elements (see Fig. 3 and Fig. S16). In contrast, some presumable RNA structures in the E1/E2 region, located at alignment positions 1430, 2574, and 2592 in the alignment (SL 1412, SL 2531, and SL 2549; JFH-1 pos. 1414, 2531, and 2549; see Figs. S7 and S8), are not well enough conserved in terms of StructConsensus (StrucC), Consensus sequence (SeqC) and the limited use of synonymous codons (SynCo) to suggest a possible function.

The above predicted well-conserved structures represent previously unrecognized RNA elements conserved in the representative HCV genomes (see Fig. 3 and Figs. S15–S17), highlighting the complexity and diversity of RNA secondary structures within this viral species. The discovery of these novel structures opens up new avenues for understanding their potential functional roles in HCV replication, translation, and pathogenesis. Further investigations are warranted to experimentally validate and explore the functional significance of these newly identified RNA secondary structures in the context of HCV biology.

### A detailed sequence and RNA secondary structure comparison reveals hints into incongruent evolution

**Figure 4:**
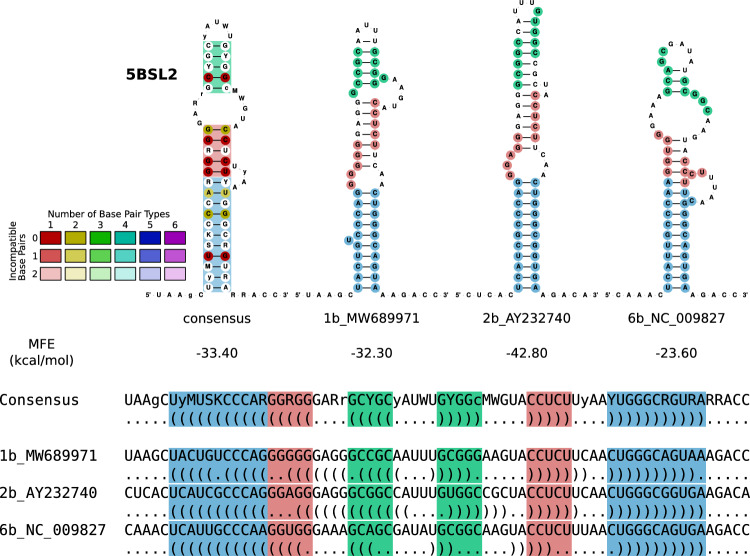
5BSL2 as an example of *incongruent evolution*. The consensus RNA secondary structure differs from the structures into which individual sequences would fold, with sequence and structure shifted relative to each other. A helix of five nucleotides (green) in the consensus is shifted by one position in MW689971 and three positions in AY232740 and three positions in NC_009827. The nucleotides that form the middle (red) stem are shifted by four nucleotides compared to the consensus.

Consensus structures are defined by base pairs that are conserved despite substitutions in the underlying sequence. In other words, base pairs that structurally correspond to each other are usually formed by pairs of nucleotides that are homologous according to their position in the sequence context. This is not always the case, however, as demonstrated by the example of the 5BSL2 stem-loop structures from three representative HCV isolates, Fig. 4. From a coarse-grained perspective, there is a consensus comprising three helical substructures. A more detailed analysis of the individual stem-loop structures, however, not only shows the expected overall conservation of the structure but also surprising differences. In addition to the expected variation, e.g., of the presence or absence of base pairs at the ends of individual helices or variations in loop sizes, we observe that the innermost helix and the hairpin loop are not formed by homologous nucleotides. Instead, a well-conserved stretch of five nucleotides forming the helix in the consensus (green) is shifted by one position in MW689971 and three positions in AY232740 and NC_009827, each. As a consequence, the terminal hairpin is conserved as a structural feature, but its individual base pairs are formed by different, non-homologous sequence positions. A similar situation is visible in the middle (red) and outer (blue) stem. The nucleotides forming the middle stem are shifted by four nucleotides relative to the consensus.

The conservation of secondary structures realized by non-homologous base pairs was termed *incongruent evolution*^70^ in contrast to the more familiar and much more frequent congruent case. Incongruent evolution can be understood as divergence of sequence alignment and structure alignment. Starting from a sequence alignment, such as the one shown on the bottom of Fig. 4, this leads to an apparently poor conservation of the structure, indicated by the white base pairs in the consensus structure. On the other hand, focusing on the structure (shown here by helices in corresponding positions) results in mismatches (indicated by the colored intervals). It has been shown in Ref.^71^ that incongruences of sequence and structure can be explained mechanistically, e.g. by flexible structural intermediates. Selection pressures that act independently, i.e., in different functional contexts, to preserve sequence and secondary structure are particularly conducive to incongruent patterns^42,72,73^ such as the ones in the 5BSL2 stem-loop structure.

## Conclusion

We presented the first comprehensive genome-wide multiple sequence alignment (MSA), incorporating computational predictions of RNA secondary structures across the entire HCV genomes (see F5–F9, and Table 1). Our selection of 57 representative genomes across the entire phylogenetic tree is based on clustering all complete HCV genomes from the BV-BRC with HDBSCAN using k-mer distributions and dimension reduction. We added manually the RefSeq sequences. The inclusion of annotations for previously identified features, such as genome annotations, secondary structures, pseudoknots, and alternative structures facilitates seamless comparisons with other research studies. By considering suboptimal structures during the predictions with LocARNA and RNAalifold, we can detect potential alternative conformations. Our in-depth analysis included conservation of the predicted RNA secondary structures, covariance in structured stem regions, and the use of nucleotides in synonymous codons. The latter output not only provides information about the overall conservation of a sequence but also information about the degree of conservation that extends beyond the requirements of the underlying amino acid sequence in coding regions. This information is important for complementing the overall degree of sequence conservation, in particular in single-stranded regions of the conserved RNA secondary structures like apical loops or bulges.

In the 5BSL2 stem-loop, we encountered examples of an incongruent mode of evolution, where sequence and structure are conserved, but the base pairs realizing the structure are not formed by homologous nucleotides. Such situations may be indicative of functionally independent selection pressures on sequence and structure^72^. As a consequence, part of the conserved structure is not detectable in a sequence-based alignment. Evolutionary incongruencies thus may reduce the sensitivity of consensus structure prediction methods. The local nature of the ‘shifts’ between sequence and structure, on the other hand, still makes it possible to detect larger structured elements, such as the 5BSL2 stem-loop, which contains a sufficient subset of congruent base pairs formed by homologous nucleotides.

All conserved RNA secondary structure models have been added to the Rfam database in version 14.10 or later. The alignment will serve as a standard for future work on HCV.

### Supplementary Information

Supplementary Information 1.

Supplementary Information 2.

## Data Availability

The alignments are available in the supplementary information (see Files F5–F9): (1) the nucleotide alignment with additional annotations such as RNA secondary structures and genes (F5); (2) the protein alignment with gene annotation (F6); and (3) the nucleotide and protein alignment combined with additional annotations such as RNA secondary structures and genes (F7). The RNA secondary structure models are provided in the Rfam database^46,47^.
