# Supplementary material for: Comprehensive survey of conserved RNA secondary structures in full-genome alignment of Hepatitis C virus
Source: Sci Rep. 2024 Jul 2;14:15145. doi: 10.1038/s41598-024-62897-0 (PMC11219754; doi:10.1038/s41598-024-62897-0)
Supplement: Supplementary file 1 — Supplementary Information 1. [file 41598_2024_62897_MOESM1_ESM.pdf]

Supplementary Information

Files

- F1 - original data set in `Fasta (fasta)` format
- F2 - pre-filtered data set in `Fasta (fasta)` format
- F3 - phylogenetic tree in `Newick (treefile)` and `Nexus (nex)` format
- F4 - representative genomes in `Fasta (fasta)` format
- F5 - nucleotide alignment in `ClustalW (clustal)`, `Fasta (fasta)`, and `Stockholm (stk)` format (latter format with additional annotations such as RNA secondary structures and genes)
- F6 - protein alignment in `ClustalW (clustal)`, `Fasta (fasta)`, and `Stockholm (stk)` format
- F7 - nucleotide and protein alignment in `Stockholm (stk)` format (with additional annotations such as RNA secondary structures and genes)
- F8 - alignment of the 5' UTR (51 sequences) in `Stockholm (stk)`, `ClustalW (clustal)`, and `Fasta (fasta)` format
- F9 - alignment of the 3' X-tail (11 sequences) in `Stockholm (stk)`, `ClustalW (clustal)`, and `Fasta (fasta)` format

Clustering

Table S1. Clustering results of the initial HCV data set using `ViralClust`[36]. #Seqs – Number of Sequences; #Clstr – Number of Cluster; minClstr – Smallest Cluster; maxClstr – Largest Cluster; avClstr – Average Cluster Size; medClstr – Median Cluster Size; #UnclstrSeqs – Number of Unclustered Sequences

Algorithm	#Seqs	#Clstr	minClstr	maxClstr	avClstr	medClstr	#UnclstrSeqs
`HDBSCAN`	2 549	36	7	583	67.42	20	121
`cd-hit-est`	2 549	105	2	580	22.32	3	205
`sumaclust`	2 549	77	2	464	23.43	3	131
`MMseqs2`	2 549	100	2	623	23.59	3	190
`vclust`	2 549	124	2	535	19.22	3	166

Genome completeness of representative genomes

- Complete Genome:
  `NC_009823.1, NC_004102.1, NC_009827.1, NC_030791.1, AB047639.1, JF735122.1, MG717928.1, AF169004.1, JF735124.1, MH427311.1, AJ851228.1, KM504115.1, KJ470619.1, KJ439768.1, MK139017.1, DQ278891.1, EU158186.1, KJ678751.1, FJ462437.1, KC248199.1, KJ439777.1, JX227965.1, AY878652.1, MG717925.1, KY348757.1, EU234061.2 , EU234065.2`
- Complete CDS:
  `NC_009824.1, NC_009825.1, NC_009826.1, NC_038882.1, KC197233.1, MN628597.1, KM043284.1, MH590700.1, AB677533.1, KC197230.1, KC844040.1, KY620874.1, KY620603.1, MK327987.1, AY232740.1, MW689975.1, MW690013.1, MW689965.1, MN977327.1, MK548369.1, MW689971.1, AY587845.1, LC435023.1, KX767023.1, MW689962.1, MW689980.1, MG878999.1, MN164860.1, MN164851.1, MN164872.1`

Alignment confirms previously predicted RNA secondary structures – Additional Information

In addition to the description of the conserved RNA secondary structures in the main text, here we would like to detail one aspect of possible alternative structures. Confirming our previous *in silico* results[10], we find the conserved capability of the IRES sequence to form a predicted alternative conformation of the SL IIId, the SL IIId*, at the base of the domain III of the IRES (Figure S2 A and B, and also see the alignment with the `StructConsensus` and `Consensus` outputs in Figure S3). Also in our RNA secondary structure alignment presented here, covering the sequence space of virtually all HCV isolates, the alternative SL IIId* structure is conserved in the predicted IRES structure with an MFE slightly lower than the IRES structure that comes with the classical SL IIId form. Even though the classical SL IIId has been repeatedly experimentally validated and shown to be functionally important[12,74–78], the structure presented here may have functional importance. This hypothesis is based on the lower MFE of this SL IIId* structure and on the following aspects. The sequence `GCGAAA` in the apical loop of the predicted alternative SL IIId* is virtually completely conserved, although this sequence is also partially single-stranded in the classical SL IIId form, a situation that likely would allow for possible sequence variation. Moreover, the five base pair stem carrying the apical `GCGAAA` loop of the SL IIId* is structurally conserved by covariations, although the classical SL IIId overlaps with that sequence, underlining the importance of the SL IIId*. Last but not least, the stem of the alternative SL IIId* appears to be slightly more stable by the number of base pairs that can be formed. We can only speculate if this alternative SL IIId* represents a structure that may be important in the IRES when not bound to ribosomes, likely after NS5B has bound the CRE/5BSL3.2 and by that interferes with the long-range interaction (LRI) between SL IIId and the bulge of the CRE, i.e. in the course of the switch from translation to replication.

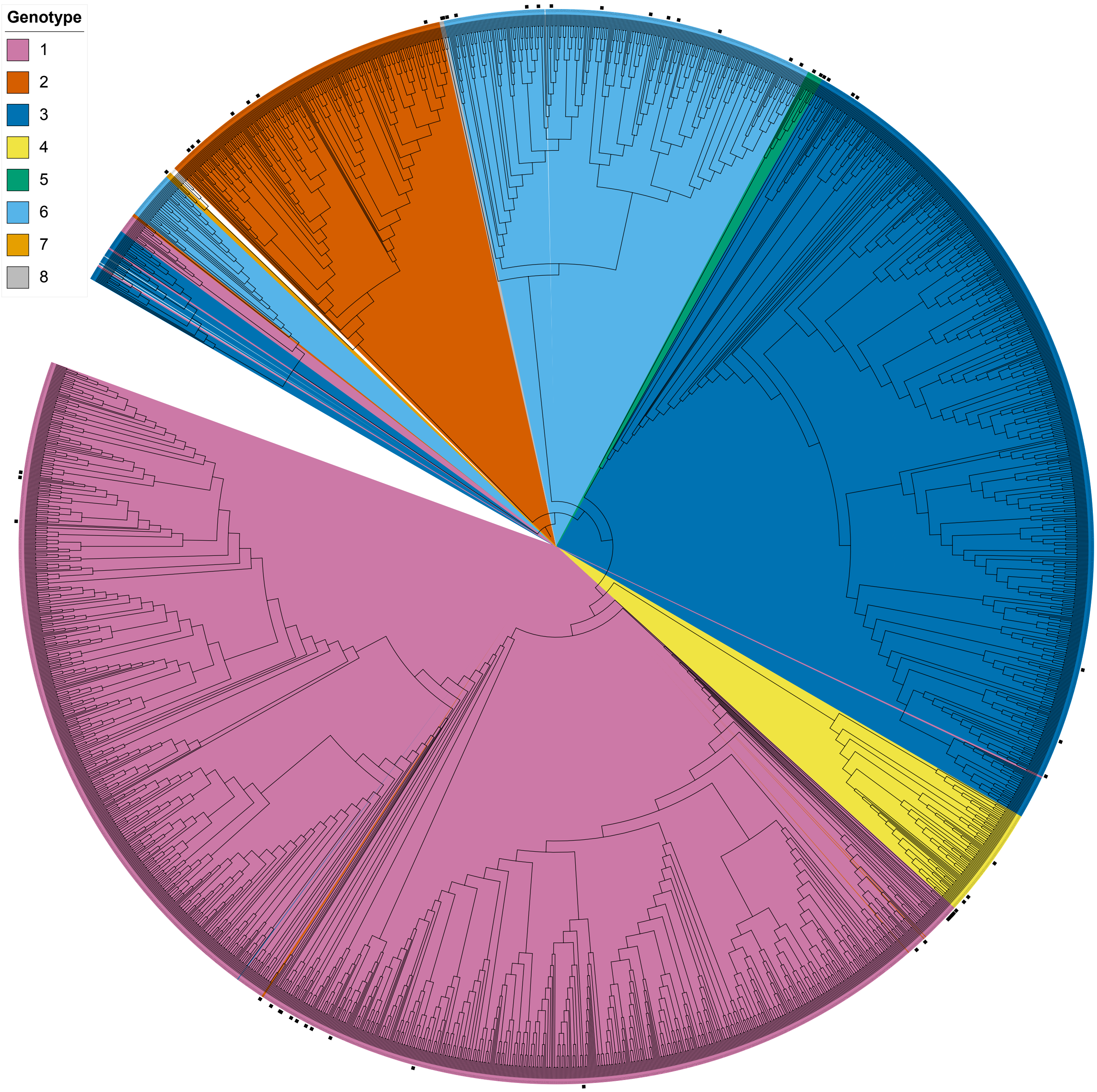

Figure S1. Phylogenetic tree of 2 549 HCV genomes obtained from `BV-BRC` database. The tree was reconstructed using `IQ-TREE 2`[79], employing an alignment generated by `MAFFT`[38]. The data set comprises all eight genotypes. While a few outliers are observed, the phylogenetic tree demonstrates clustering patterns corresponding to the different genotypes. The 57 representative genomes used for alignment construction are labeled with black boxes.

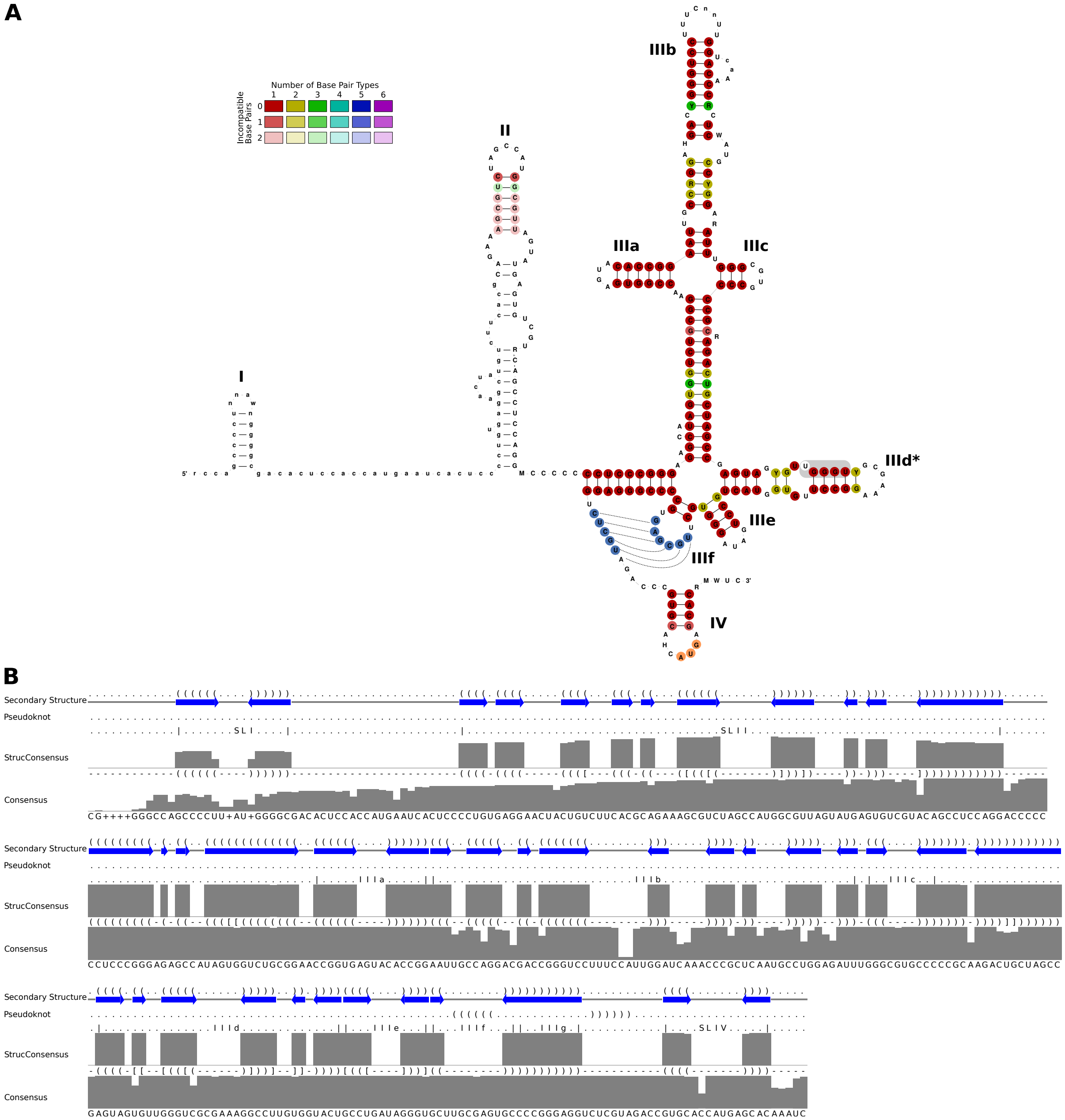

Figure S2. Conservation of the HCV 5' UTR. (A) Alignment-based RNA secondary structure prediction of the 5' UTR. Among the selected sequences, 51 isolates had a 5' UTR sequence (please see the additional alignment of only 5' sequences in Figure S3 and F8). The HCV IRES comprises the stem-loops (SLs) II to IV, with the polyprotein start codon located in the loop of SL IV. The IRES structure shows the same alternative structure of SL IIId* as in[10] due to the slightly lower MFE (-116.90 kcal/mol) compared to the 'classical' experimentally validated structure. The sequence `UGGGU` that would be in the apical loop of the classical form of SL IIId is marked by the gray box. Only the nucleotides involving the loop of SL IIIf and part of the single-stranded stretch upstream of SL IV were manually manipulated to form a pseudoknot (blue), while the sequences involved in this pseudoknot actually were predicted to be single-stranded in the structure alignment. A low degree of covariance (see color code) suggests that not only the RNA secondary structure shown here is important but there may be also a requirement for overlapping *cis*-elements. The start codon (`AUG`) is marked in orange. The lower parts of the SL II appear largely white because on 41 sequences contain the stem-loop. (B) Sequence and RNA secondary structure features as in Figure 1 B.

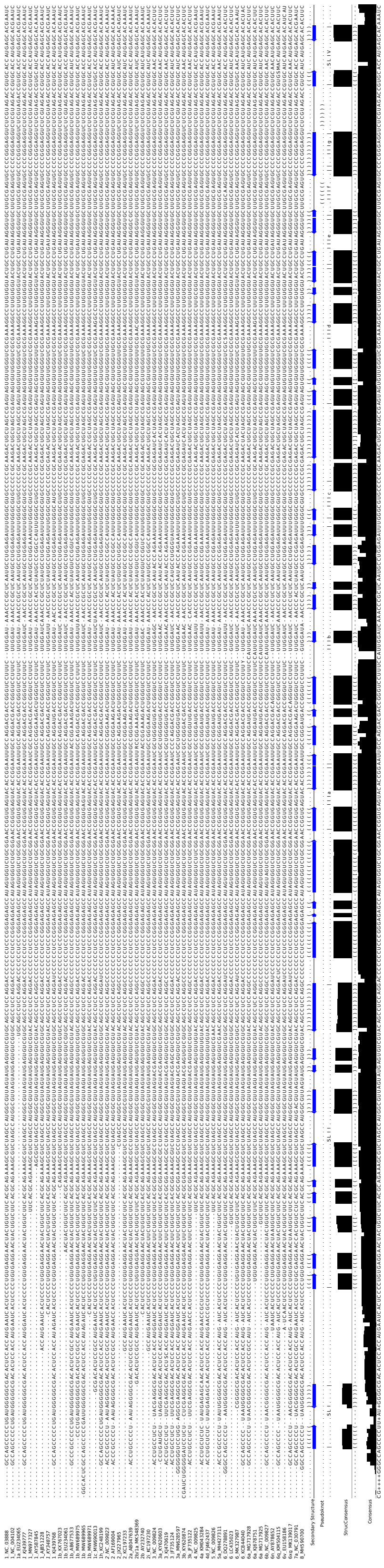

Figure S3. (related to Figure S2) Nucleotide alignment and additional sequence and RNA secondary structure features as in Figure 2 C of the 51 sequences fully covering the 5' UTR.

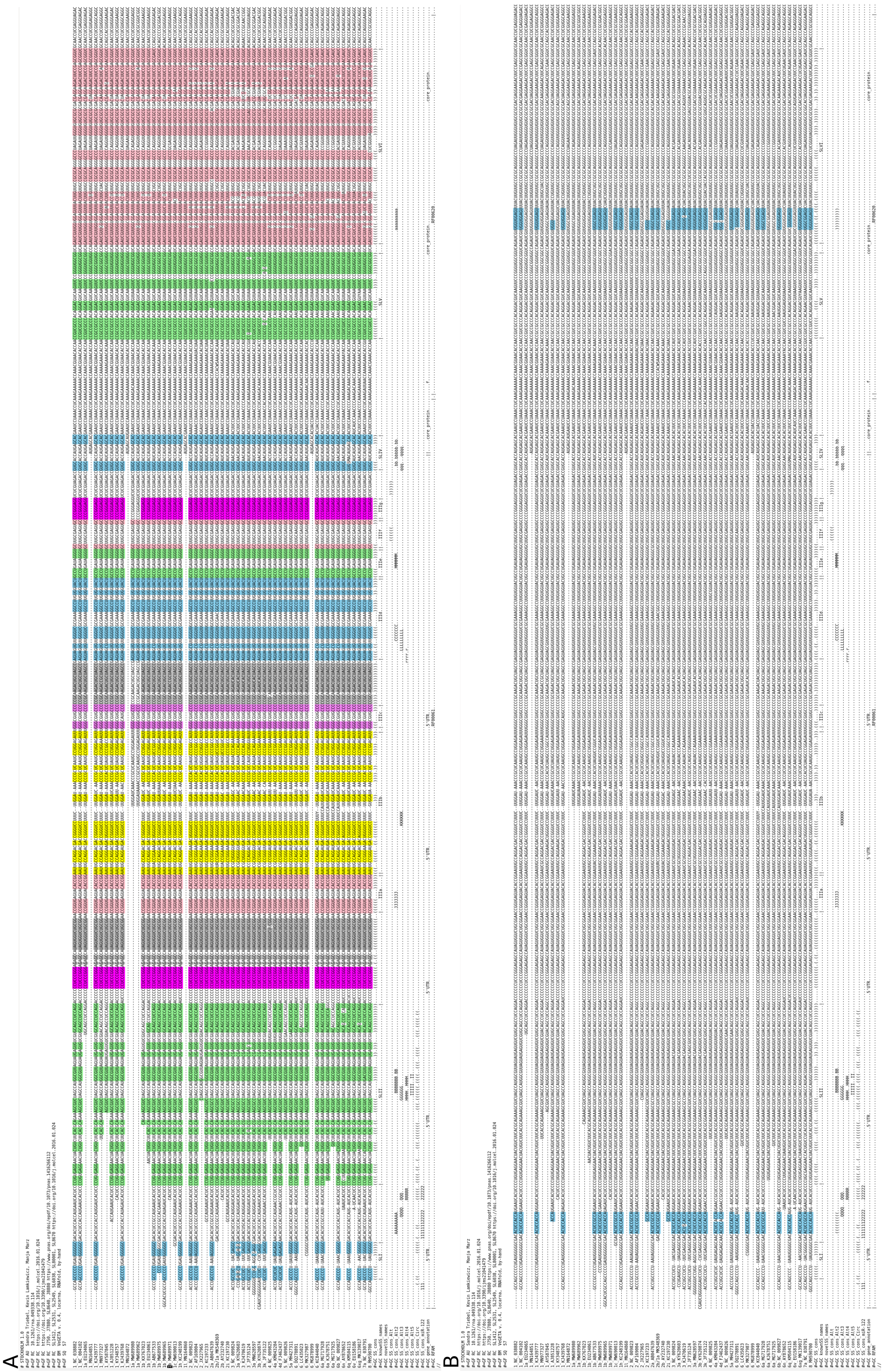

Figure S4. (Nucleotide alignment of HCV 5’ UTR and the start of the core gene of the 51 sequences fully covering the 5’ UTR show in `Emacs` in `RALEE` mode[49]. (A) Alignment coloured according to RNA secondary structures SLI, IRES, SLV, and SLVI. (B) Alignment coloured according to the interaction of interdomain I and II with the left strand of domain VI.

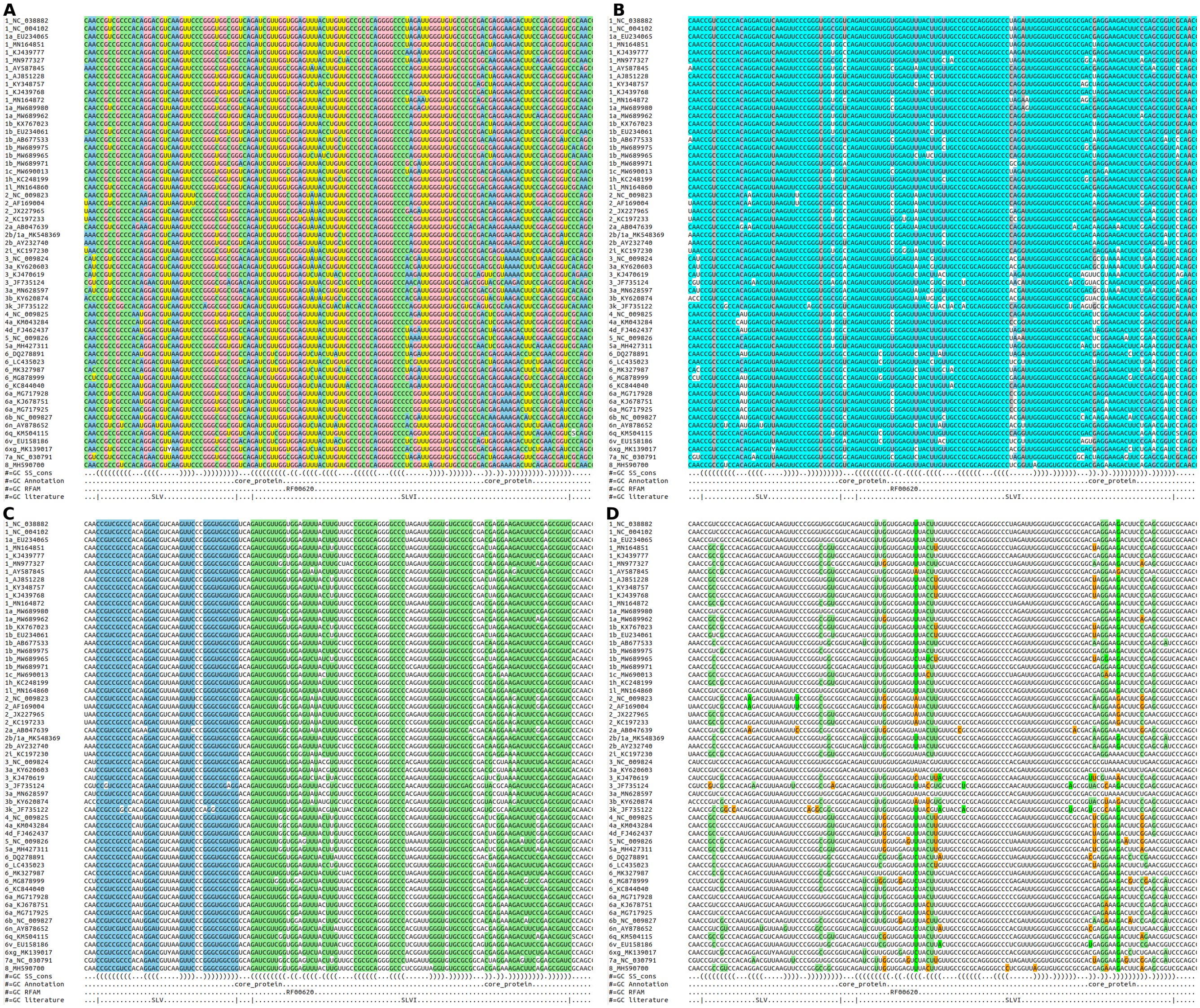

Figure S5. Nucleotide alignment visualization using `Emacs` in `RALEE` mode[49] displaying (A) nucleotide identity, (B) sequence conservation, (C) consensus RNA secondary structure, and (D) compensatory mutations.

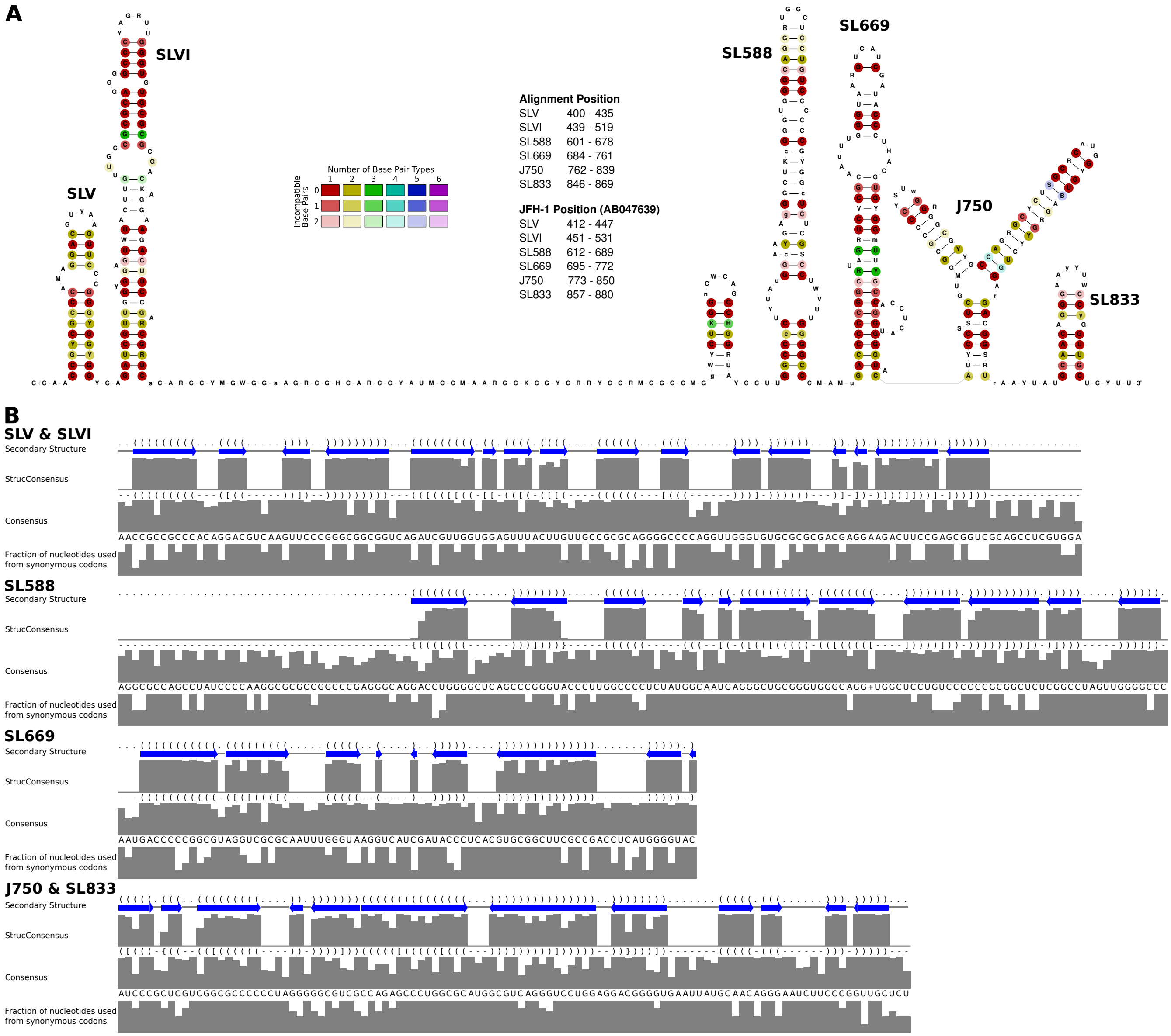

Figure S6. Conserved RNA secondary structures in the core coding region. (A) Structures shown with base color code illustrating the extent of covariations in double-stranded regions like in Figure S2. (B) Sequence and RNA secondary structure features as in Figure 1 B.

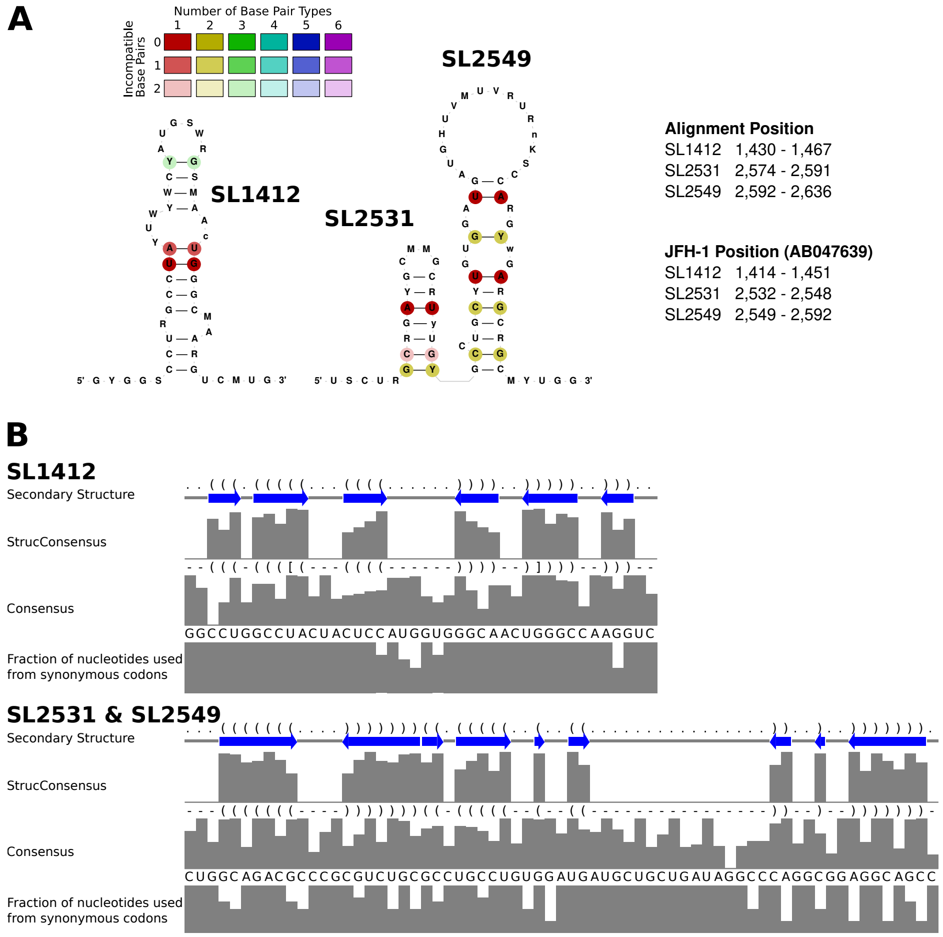

Figure S7. (A) Possible RNA secondary structures in the E1 and E2 region with its structural representation in and (B) additional sequence and RNA secondary structure features as in Figure 1 B.

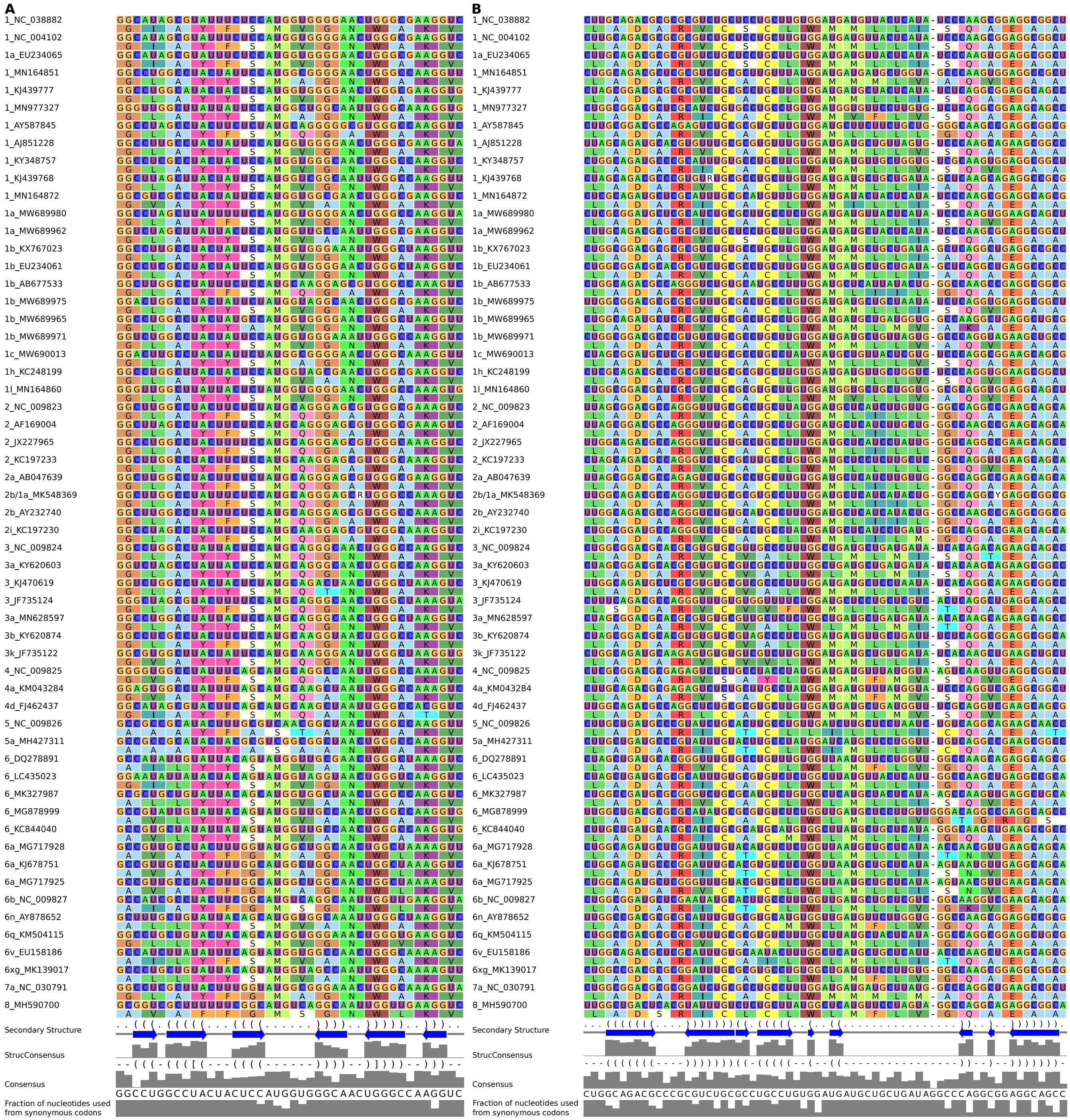

Figure S8. (related to Figure S7) Alignment of nucleotides and proteins combined and additional sequence and RNA secondary structure features as in Figure 1 B for (A) SL 1412 and (B) SL 2531 and SL 2549.

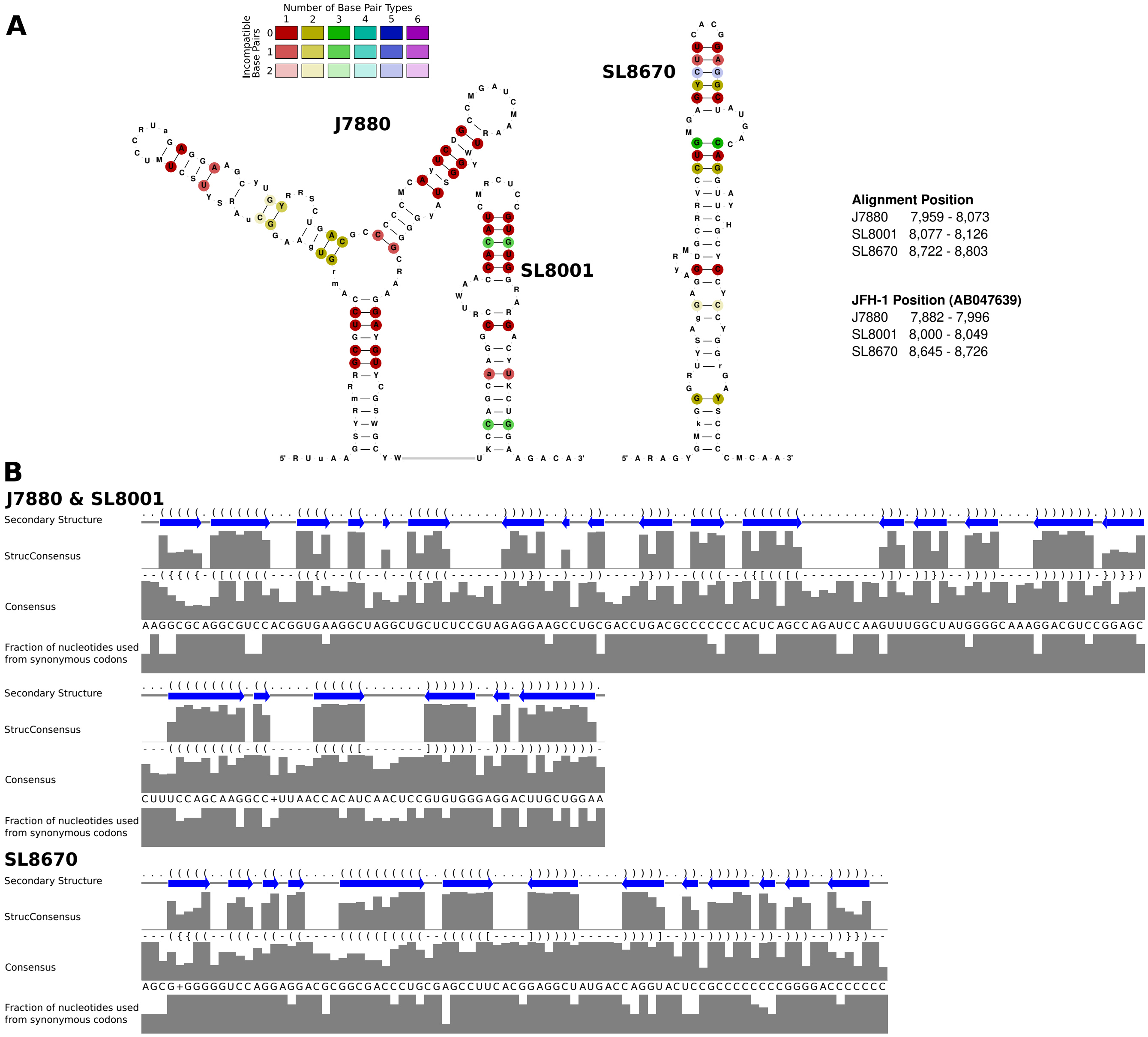

Figure S9. (A) Known RNA secondary structures in the upstream NS5B coding region and additional sequence and (B) RNA secondary structure features as in Figure 1 B.

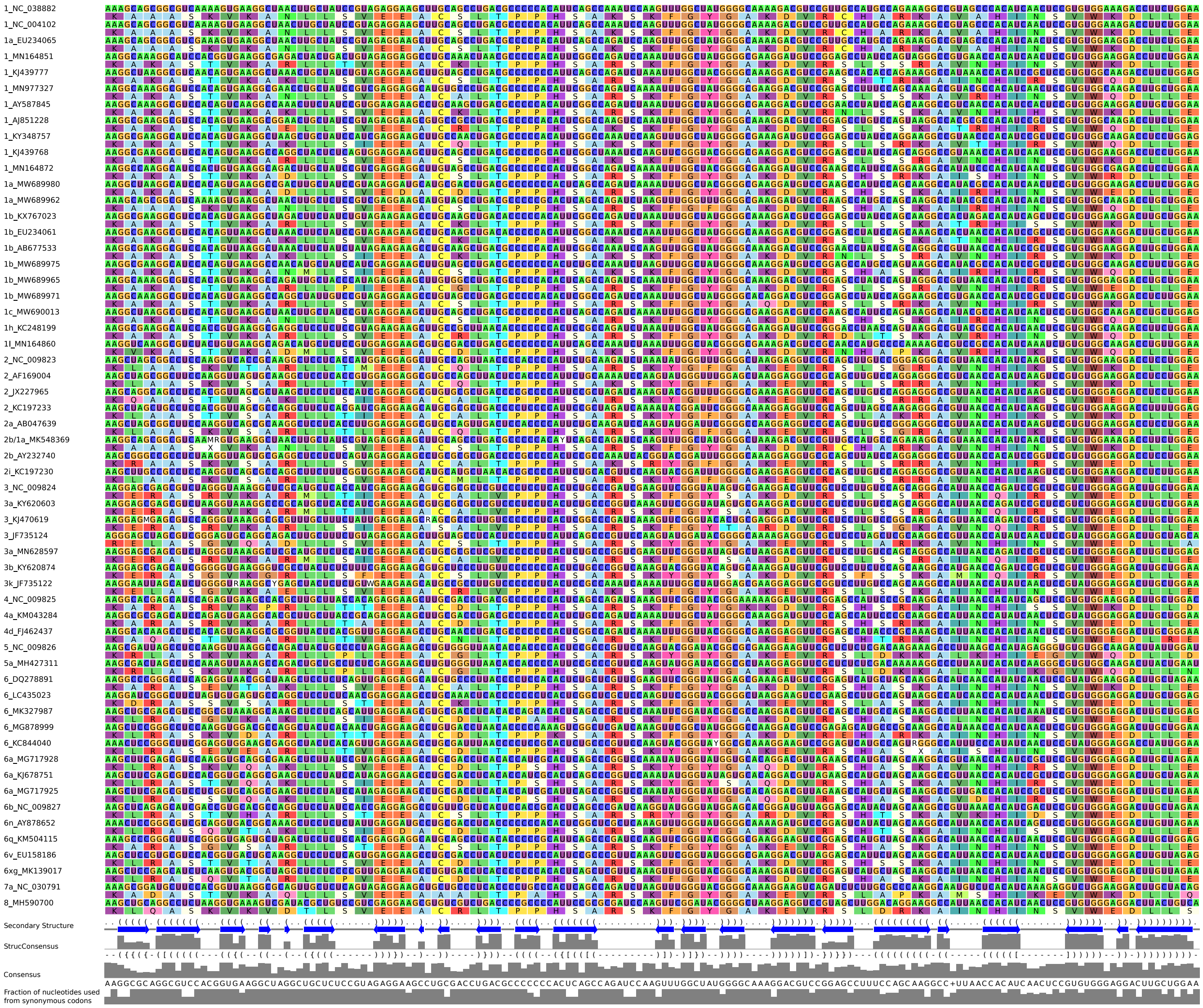

Figure S10. (related to Figure S9) Alignment of nucleotides and proteins combined and additional sequence and RNA secondary structure features as in Figure 1 B for J 7880 and SL 8001.

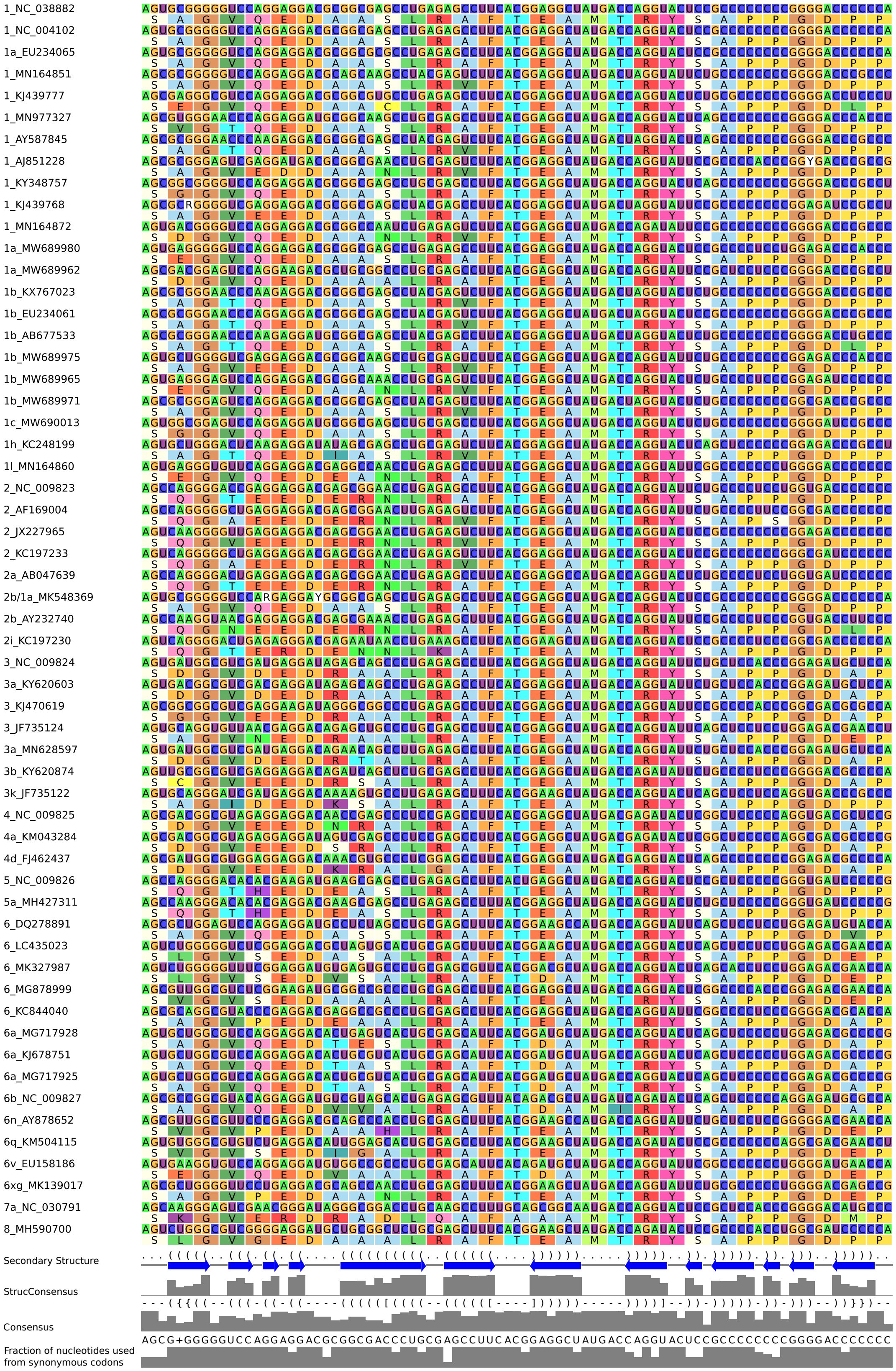

Figure S11. (related to Figure S9) Alignment of nucleotides and proteins combined and additional sequence and RNA secondary structure features as in Figure 1 B for SL 8670.

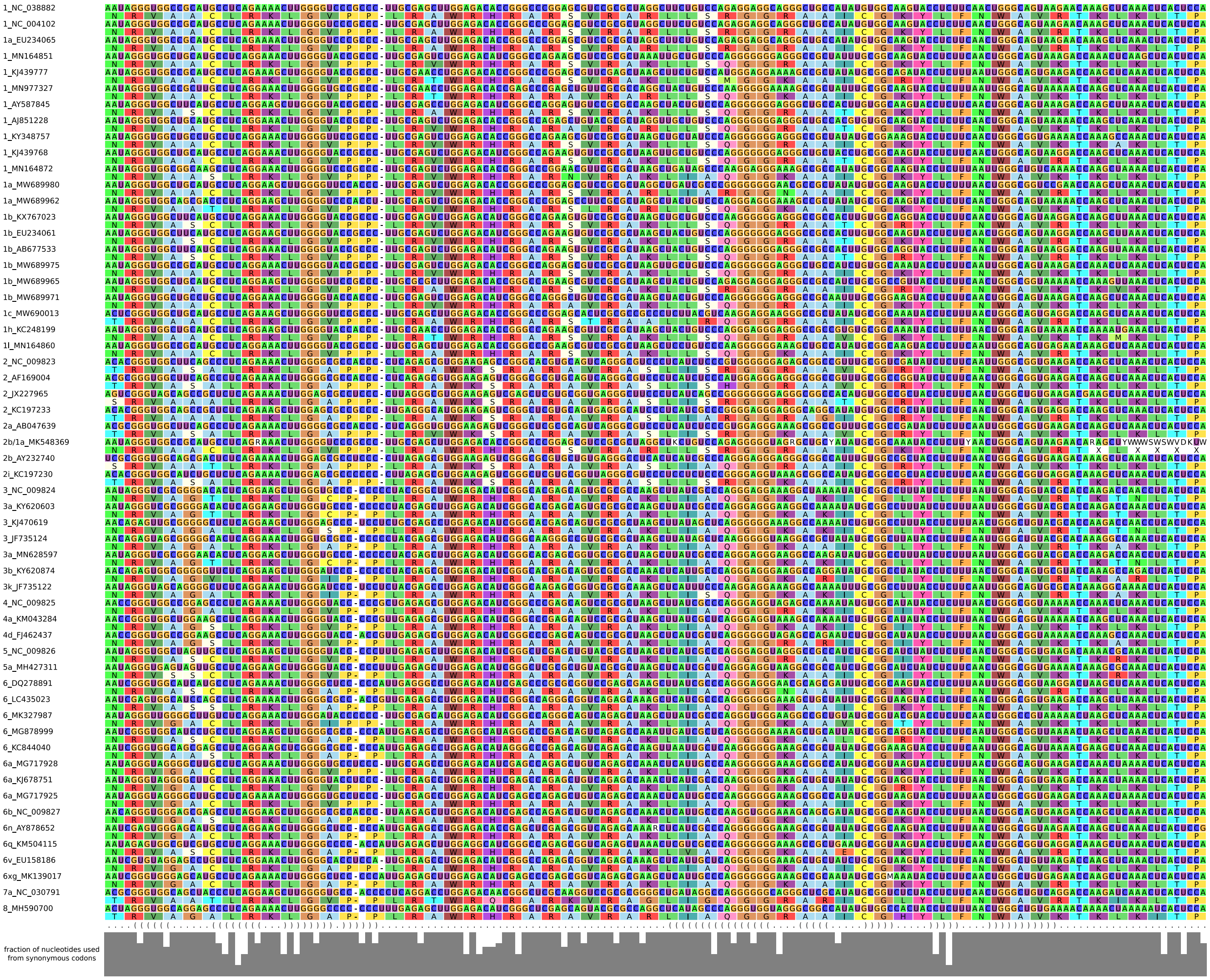

Figure S12. (related to Figure 1) Alignment of nucleotides and proteins combined and additional sequence and RNA secondary structure features as in Figure 1 B for 5BSL1 and 5BSL2.

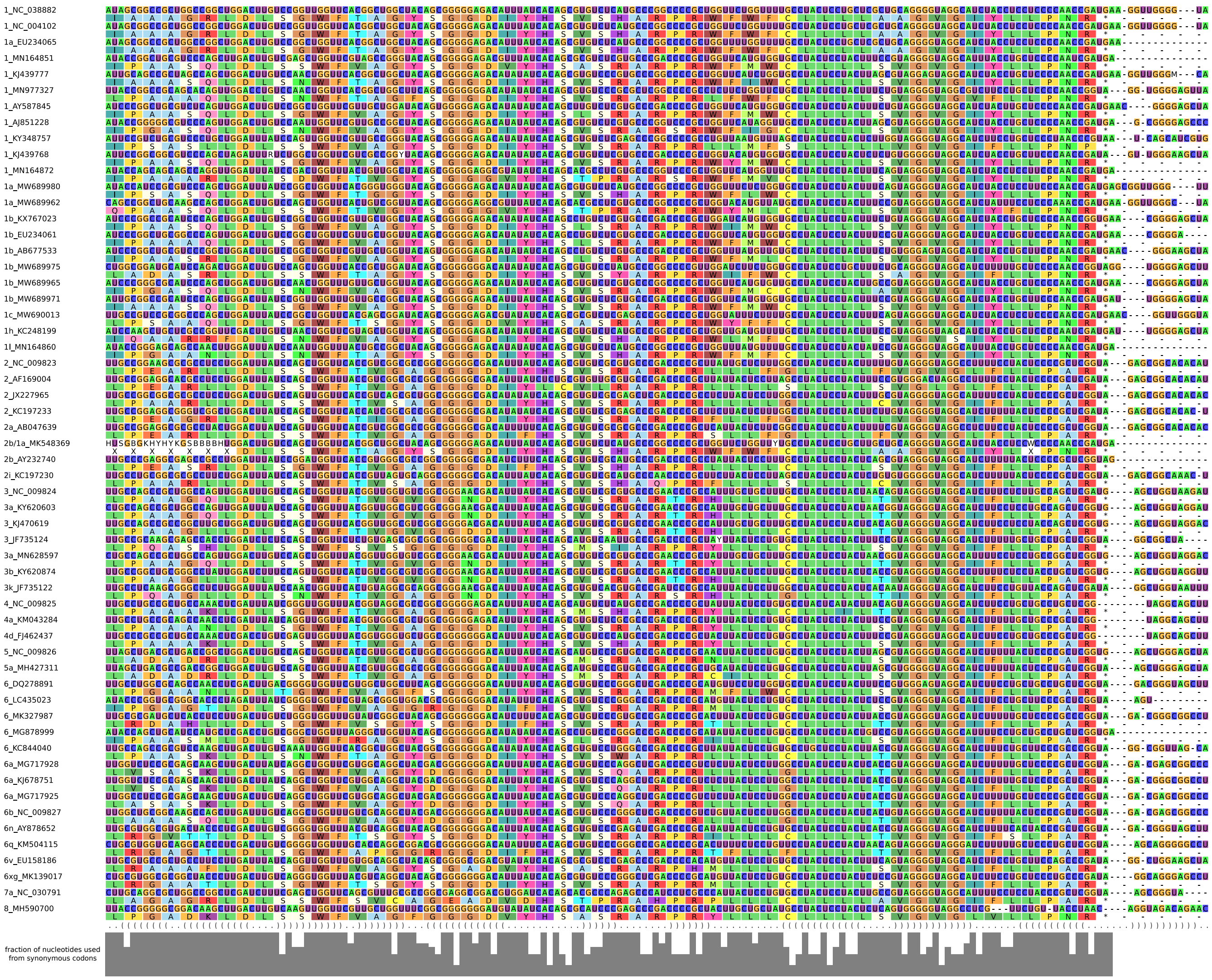

Figure S13. (related to Figure 1) Alignment of nucleotides and proteins combined and additional sequence and RNA secondary structure features as in Figure 1 B for *cis*-replication element (CRE, 5BSL3.2), 5BSL3.3 and SL IV.

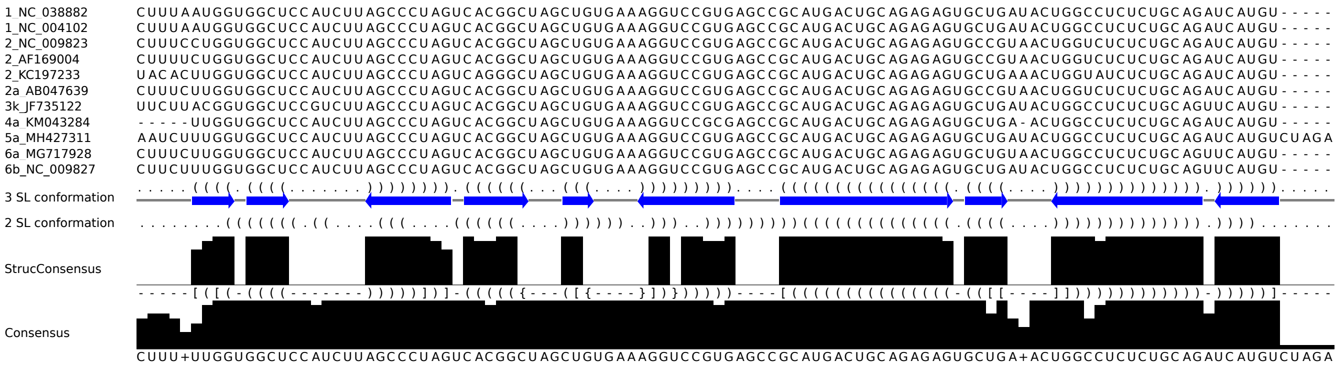

Figure S14. (related to Figure 2) Nucleotide alignment and additional sequence and RNA secondary structure features as in Figure 2 C of the 11 sequences fully covering the 3’ X region.

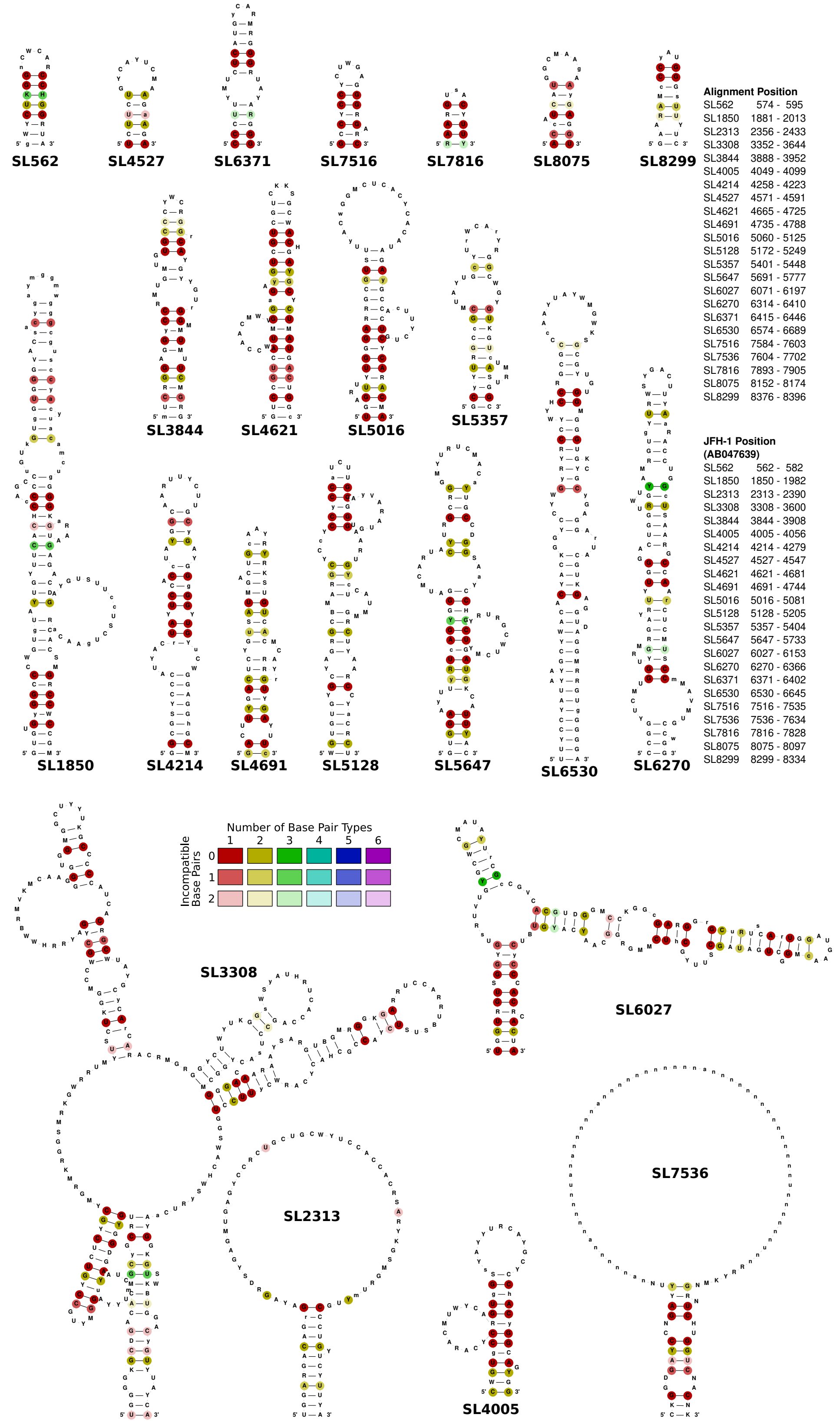

Figure S15. 23 novel conserved RNA secondary structure candidates from coding regions of the HCV alignment. RNA secondary structures are colored as in Figure 1 A.

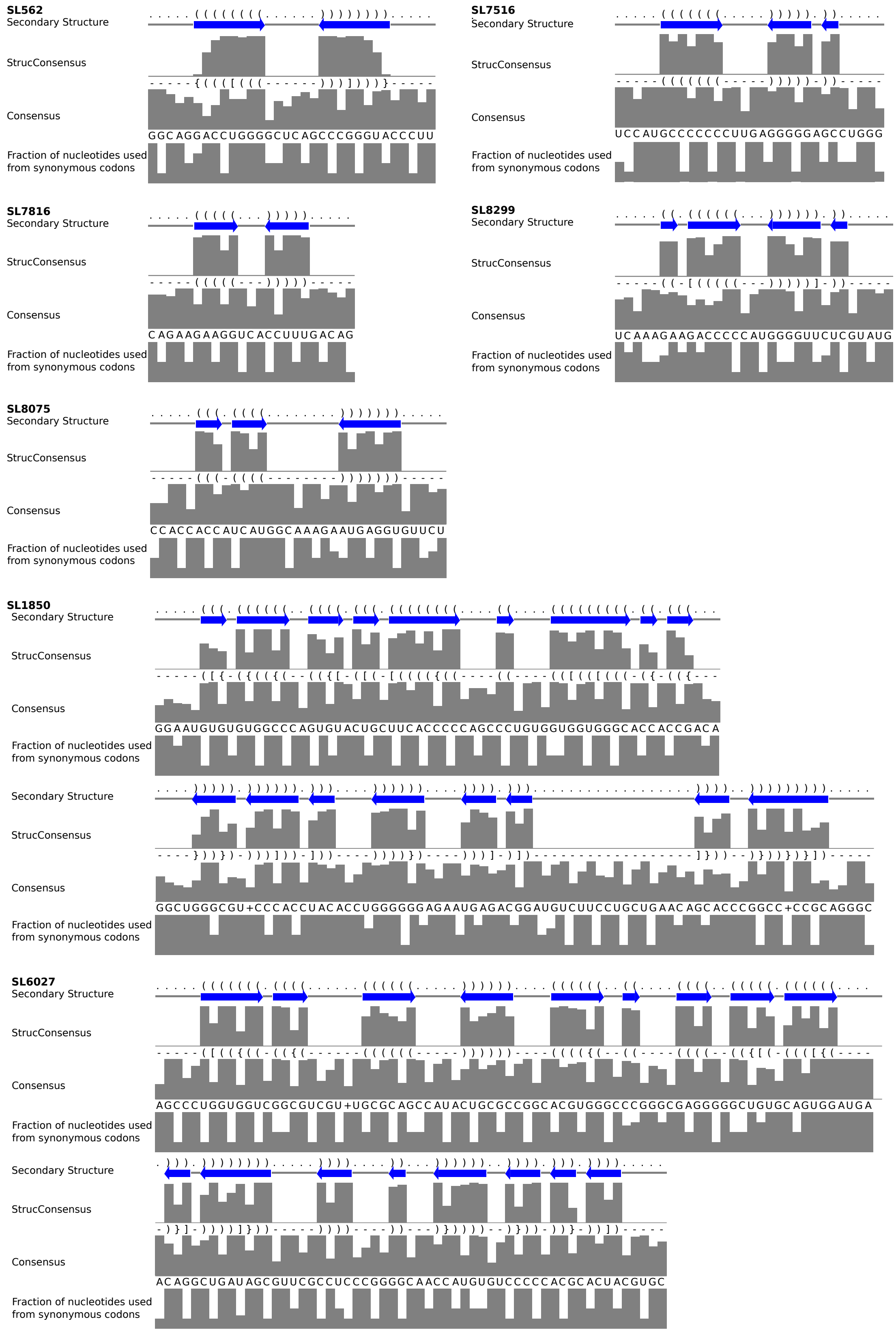

Figure S16. (related to Figure 3) Additional sequence and RNA secondary structure features as in Figure 1 B for seven novel conserved RNA secondary structure candidates.

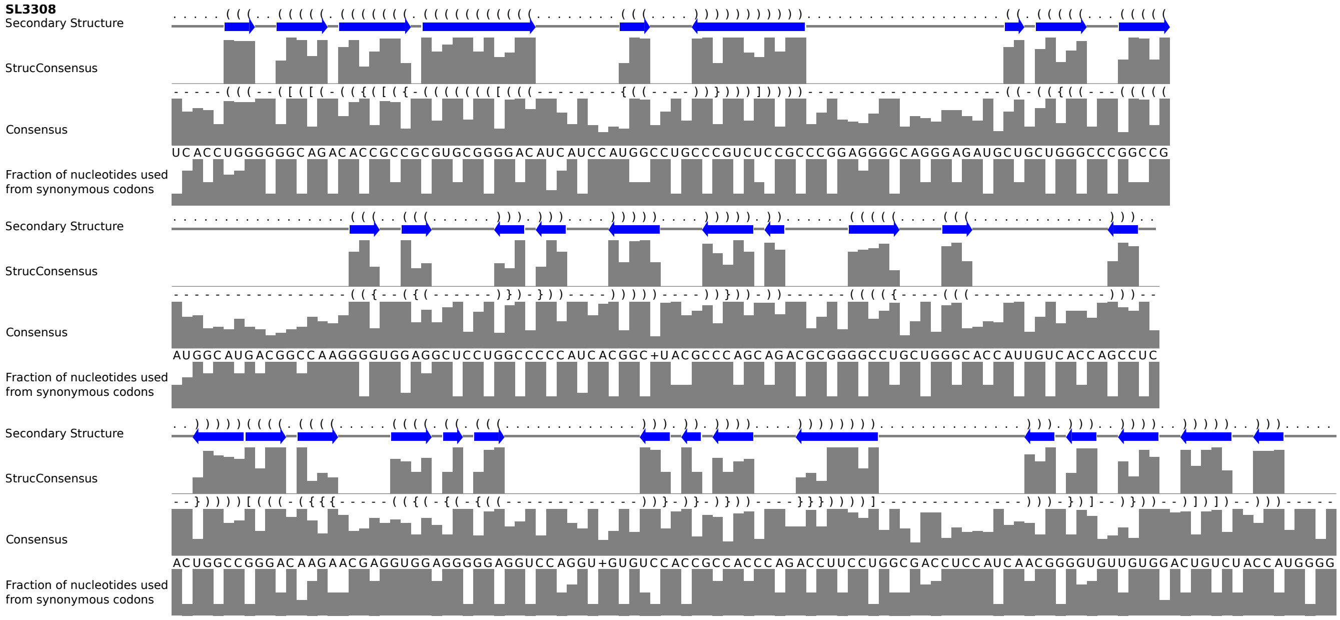

Figure S17. (related to Figure 3) Additional sequence and RNA secondary structure features as in Figure 1 B for SL 3308 as novel conserved RNA secondary structure candidate.
